# Predictors of outcome in cryptoglandular anal fistula according to magnetic resonance imaging: A systematic review

**DOI:** 10.1002/hsr2.1354

**Published:** 2023-06-22

**Authors:** Alireza Teymouri, Amir Keshvari, Ali Ashjaei, Seyed Mohsen Ahmadi Tafti, Faeze Salahshour, Faezeh Khorasanizadeh, Amirhosein Naseri

**Affiliations:** ^1^ Department of Colorectal Surgery Imam Reza Hospital Aja University of Medical Sciences Tehran Iran; ^2^ Department of Surgery Colorectal Research Center, Imam Hospital Complex Tehran University of Medical Sciences Tehran Iran; ^3^ Department of Surgery Besat Hospital Aja University of Medical Sciences Tehran Iran; ^4^ Department of Radiology Advanced Diagnostic and Interventional Radiology Research Center (ADIR) Tehran University of Medical Sciences Tehran Iran

**Keywords:** Anal fistula, magnetic resonance imaging, treatment outcomes, recurrence

## Abstract

**Background and Aims:**

Anal fistula (AF) with cryptoglandular origin tends to recur, and multiple risk factors are implicated. Recently, some magnetic resonance imaging (MRI) findings with predictive value for disease outcomes have been proposed. These intrinsic anatomic features include those of the AF and its surrounding structures. This study aims to clarify the prognostic role of MRI in AF.

**Methods:**

We performed a systematic search of PubMed, Embase, and EBSCO databases. Two independent reviewers conducted the search and screened the articles. We selected studies that used MRI to assess AF and reported its relationship to disease outcome. We extracted data regarding the study design, type of intervention, outcome, MRI‐measured items, and their significance.

**Results:**

Out of 1230 retrieved articles, 18 were eligible for final inclusion, and a total of 4026 patients were enrolled in the selected studies. For preoperative MRI, the significant items affecting the outcome were the length of the fistula, horseshoe type, presence of multiple tracts, supralevator extension, and apparent diffusion coefficient (ADC) value. Other studies investigated the healing process using postoperative MRI.

**Conclusion:**

This review found that MRI can be useful in the management of AF, both preoperatively and postoperatively. Factors, such as fistula length, horseshoe type, presence of multiple tracts, supralevator extension, and ADC value were found to be significantly associated with treatment outcomes. The presence of the fistula tract and the development of new abscesses on postoperative MRI was found to hinder the healing process. Further studies are needed to confirm these findings.

## 1 INTRODUCTION

Fistulas are abnormal tracts or cavities that connect two epithelialized surfaces, usually between the mucosal surfaces and the skin. Anorectal or anal fistula (AF) consists of the tract(s), and internal and external openings and affect the anal canal and the perineum.^1^, ^2^ The success rate for different surgical and nonsurgical options for AF treatment varies, and failure is common. Thus, patients and treating clinicians may struggle with recurrent fistula. Recurrence happens when the fistula reappears within 1 year after the surgical intervention.^3^, ^4^ Across the literature, the reported recurrence rates range between 5% and 69% with cutting seton fistulotomy and fibrin sealants injections, respectively.^5^, ^6^ Several risk factors contribute to the recurrence of fistula. Examples of these risk factors include high daily salt intake, metabolic syndrome, prior surgery, and a sedentary lifestyle.^7^

Diagnosis is based on history and physical examination. The external opening and drainage might be visible in physical examination.^8^ The anatomic relation of the fistula to the surrounding structures is confirmed with preoperative imaging techniques, such as endoanal ultrasound, computerized tomography scan, and especially magnetic resonance imaging or MRI.^9^, ^10^ Several treatment options with varying success rates are available for AF. The main treatment options are as follows: fistulotomy (the mainstay of therapy for simple fistulas), fistulectomy, fibrin glue injection, ligation of the intersphincteric fistula tract (LIFT procedure), and the advancement flap.^11^, ^12^ Treatment goals are mainly directed at wound healing, prevention of recurrence, and incontinence.^13^

Regarding the prognosis, MRI is useful in two ways: First, it most accurately demonstrates the anatomic location of fistula components and determines the accompanying structures, such as abscesses.^14^, ^15^ Second, MRI is more feasible to assess the fistula's inflammatory activity which is probably associated with postoperative recurrence.^16^ As stated before, preoperative MRI is done in quite a few patients with AF to aid in diagnosis and treatment. Few studies have directly investigated its role in predicting the long‐term outcome of treatment using both anatomic items and fistula activity.^16^, ^17^, ^18^ Furthermore, less information is available regarding the predictive value of postoperative MRI.^19^ Hitherto, to our knowledge, there is no existing systematic review to assess and compare the findings of these studies altogether. Thus, this study is designed to clarify MRI's prognostic role in AF.

## 2 METHODS

### 2.1 Study design

We conducted the present systematic review according to the Preferred Reporting Items for Systematic Reviews and Meta‐Analysis (PRISMA) guideline. The review protocol is registered in PROSPERO and is available online (CRD42022385181). A literature search was performed in PubMed and Embase, and EBSCO databases with the search terms “rectal fistula” in combination with “treatment outcome” in combination with “Magnetic resonance imaging” from inception to February 2023. We used Medical Subject Headings terms in PubMed, Emtree terms in Embase, and other keywords to find the relevant papers as outlined in Appendix. The search results were not restricted by date but were limited to the English language. We also did a complementary search in Google Scholar and hand searched the references of each selected study to avoid missing potentially relevant articles for this review.

### 2.2 Eligibility criteria and quality assessment

Prospective or retrospective studies that reported the association between MRI‐measured items and treatment outcome/failure in cryptoglandular AF were selected for final evaluation. The studies that were primarily concerned with fistulizing Crohn's disease, various malignancies, animal models, use of ultrasound in the AF, and rectovaginal fistula were discarded. Also, abstracts, reviews, ongoing studies, commentaries, letters, and editorials were excluded. We used the Newcastle‐Ottawa quality assessment form for cohort studies to evaluate the selected papers. Accordingly, qualified articles had to have fair and good quality.^20^

### 2.3 Screening of the studies

The results of our preliminary search were assessed independently by two evaluators. First, we did a search for the duplicates and evaluated the studies by title according to inclusion and exclusion criteria. Afterward, the selected articles were assessed based on the abstract, and in case of disagreement, full‐text articles were retrieved and examined. We contacted the corresponding author for a copy if the full text was unavailable. If needed, a decision was made by consensus or by a third reviewer. We extracted the study design, study set, total number of patients, type of intervention, and follow‐up period in each study. Also, the studied outcome(s), MRI‐measured items, and the significance of their impact on the outcome were extracted. A *p* < 0.05 was considered significant.

## 3 RESULTS

**Figure 1:**
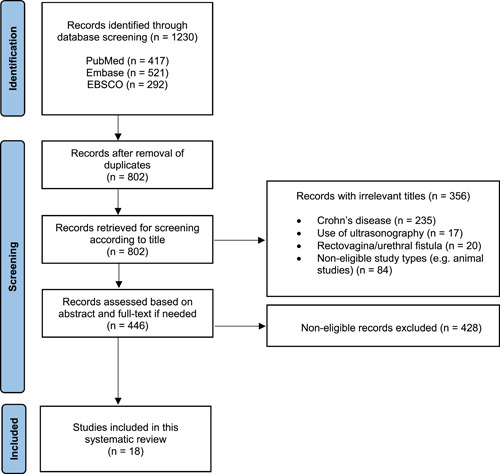
Preferred Reporting Items for Systematic Reviews and Meta‐Analyses flow diagram of the study selection process.

Our systematic search yielded 1230 articles of which 428 were duplicates, and thus were eliminated. Articles directly addressing fistulizing Crohn's disease accounted for 235. The rest of the articles were evaluated based on title and abstract, and full‐text if needed. Finally, a total of 18 studies met the inclusion criteria and were retrieved for final analysis. The PRISMA flow diagram of the study selection process is shown in Figure 1. Of these, 13 were retrospective, 4 were prospective cohort, and 1 was a case‐control study. Overall, 14 studies dealt with preoperative MRI, whereas 4 studies were about postoperative MRI findings. The pooled number of patients was 4026, and they underwent different procedures including surgery, collagen paste, advancement flap, the LIFT, LASER therapy, and fibrin sealant. The follow‐up period ranged from 1 to 151 months. The information about the study design and its characteristics are summarized in Table 1.

**Table 1:** Study design and characteristics including type of intervention, total number of patients, and follow‐up period.

ID	Study	Country	Study design	Total number of patients	Type of intervention	Follow‐up period (months)	NOS score
1	Mitalas et al.^21^	The Netherlands	Retrospective cohort	162	Advancement flap	12	7
2	van Onkelen et al.^22^	The Netherlands	Retrospective cohort	252	Advancement flap	6–136	6
3	Garg et al.^23^	India	Prospective cohort	51	Surgery	5–14	6
4	Giordano et al.^24^	United Kingdom, Italy, Denmark	Prospective cohort	100	Collagen paste	12	5
5	Liu et al.^16^	China	Retrospective cohort	82	Surgery	24	6
6	Raja et al.^25^	India	Retrospective cohort	35	Surgery	12	7
7	Usta^26^	Turkey	Retrospective cohort	98	Surgery	33	7
8	Garg et al.^27^	India	Retrospective cohort	1250	Surgery	1–151	8
9	Garg et al.^28^	India	Retrospective cohort	325	Surgery	7–67	7
10	Garg et al.^29^	India	Retrospective cohort	757	Surgery	4–84	7
11	Sarmiento et al.^18^	United States	Retrospective cohort	22	LIFT	N/A	5
12	Darwish et al.^30^	Egypt	Case‐control	100	LASER/fistulotomy	12	6
13	Hegab et al.^31^	Egypt	Prospective cohort	30	LIFT	24	7
14	Yagnik et al.^32^	India	Retrospective cohort	182	Surgery	4–73	6
15	Buchanan et al.^33^	United Kingdom	Prospective cohort	22	Fibrin sealant	2–24	7
16	Garg et al.^34^	India	Retrospective cohort	325	Surgery	14–68	6
17	Dawka et al.^35^	India	Retrospective cohort	50	Surgery	12–20	7
18	Garg et al.^36^	India	Retrospective cohort	183	Surgery	12–48	7

Abbreviations: LIFT, ligation of intersphincteric fistula tract; NOS, Newcastle‐Ottawa Scale; N/A, not available.

The relationship between the following items and outcome was investigated in the selected studies with preoperative MRI: 1. AF type (i.e., intersphincteric, transshpincteric, suprasphincteric, extrasphincteric, and horseshoe type fistula), 2. Anatomic features of internal opening (i.e., its location, number, and nondetection), 3. Characteristics of fistula tract (i.e., multiple tracts, course of the tract, and its length), and 4. Other factors include abscess, supralevator extension, sphincter involvement, and the apparent diffusion coefficient or ADC value. Of these, fistula's length, horseshoe type, presence of multiple tracts, supralevator extension, and ADC value were significantly associated with the reported outcome (*p* < 0.05). These findings are summarized in Table 2. Of 18 studies, only 4 investigated the impact of postoperative MRI on AF outcome. All of these studies assessed the disease activity at 3 months after the surgery. The presence of the fistula tract on postoperative MRI, healing of internal opening, healing of intersphincteric tract, healing of external tracts, and the development of new abscess, which affect the healing process were the studied items, as shown in Table 3.

**Table 2:** Summary of the studied items by MRI in selected studies.

Feature	Details
Type of fistula	1. Intersphincteric 2. Transshpincteric 3. Suprasphincteric 4. Extrasphincteric 5. Horseshoe type^a^
Internal opening	1. Number of IO 2. Nondetection of IO 3. Location of IO
Fistula tract	1. Multiple tracts^a^ 2. Tract type (linear, curvilinear) 3. Length, width, depth
Other features	1. Abscess 2. Supralevator extension^a^ 3. Sphincter involvement 4. ADC value^a^
Postoperative MRI findings	1. Presence of fistula tract 2. Healing of IO 3. Healing of intershphincteric tract 4. Healing of external tracts 5. Development of a new abscess

Abbreviations: ADC, apparent diffusion coefficient; IO, internal opening; MRI, magnetic resonance imaging.

^a^ Predictors with statistical significance.

**Table 3:** Treatment outcomes in the included studies, and significant MRI‐measured items.

ID	Study	Studied outcome(s)	Reported items (MRI)	Significant items	*p* Value, odds ratio
1	Mitalas et al.^21^	Healing	1. Direct course versus horseshoe 2. Abscess	Direct course	0.001^a^
2	van Onkelen et al.^22^	Failure	1. Abscess 2. Location of IO 3. Horseshoe extension	No horseshoe extension	0.005^a^, 1.8
3	Garg et al.^23^	Healing	1. Multiple tract 2. Horseshoe fistula 3. Abscess 4. Anterior tract 5. Nondetection of IO	None	N/A
4	Giordano et al.^24^	Healing	1. Park's classification 2. Length of the fistula tract	Fistula length	0.03^b^, 0.742
5	Liu et al.^16^	Recurrence	1. Park's classification 2. IO (number) 3. Abscess 4. Multiple tracts 5. ADC value	Multiple tracts ADC value (in patients with abscess)	0.05^a^ 0.03^b^
6	Raja et al.^25^	Recurrence	1. Park's classification 2. Type of tract (e.g., linear and curvilinear) 3. Supralevator extension	Supralevator extension	0.001^a^
7	Usta^26^	Recurrence	1. Abscess 2. Park's classification 3. SJUH classification 4. Number of tracts	None	N/A
8	Garg et al.^27^	Healing	Abscess	None	N/A
9	Garg et al.^28^	Healing	Abscess	None	N/A
10	Garg et al.^29^	Healing	Nondetection of IO	None	N/A
11	Sarmiento et al.^18^	Recurrence	1. Length of AF 2. Width of AF 3. Depth of AF	None	N/A
12	Darwish et al.^30^	Recurrence	1. Fistula length 2. Sphincter affection	None	N/A
13	Hegab et al.^31^	Recurrence	Fistula length	Fistula length	<0.05^b^
14	Yagnik et al.^32^	Healing	Nondetection of IO	None	N/A
**ID**	**Study**	**Studied outcome(s)**	**Studied items**	**SN, SP**	**PPV, NPV**
15	Buchanan et al.^33^	Recurrence	Fistula tract presence	100, 100	100, 100
16	Garg et al.^34^	Healing/recurrence	1. Healing of intersphincteric tract 2. Healing of IO	N/A	99.2, 100
17	Dawka et al.^35^	Healing	1. Healing of IO 2. Healing of intersphincteric tract 3. Healing of external tracts 4. Development of a new abscess	88.6, 100	100, 78.9
18	Garg et al.^36^	Healing	1. Healing of IO 2. Healing of intersphincteric tract 3. Healing of external tracts 4. Development of new abscess	93.9, 94.7	98.2, 83.7

Abbreviations: ADC, apparent diffusion coefficient; AF, anal fistula; IO, internal opening; MRI, magnetic resonance imaging; NPV, negative predictive value; N/A, not applicable (available); PPV, positive predictive value; SJUH, St James University Hospital; SN, sensitivity; SP, specificity.

^a^ The Pearson's *χ* ^2^ test was used to calculate these *p* values.

^b^ The Student's *t* test was used to calculate the marked *p* values.

## 4 DISCUSSION

The present review aimed to investigate the impact of preoperative and postoperative MRI on the outcome of cryptoglandular AF. The review included 18 studies, with a pooled number of 4026 patients who underwent different procedures, such as surgery, collagen paste, advancement flap, LIFT, LASER therapy, and fibrin sealant, with a follow‐up period ranging from 1 to 151 months. The results showed that fistula's length, horseshoe type, presence of multiple tracts, supralevator extension, and ADC value were significantly associated with the reported outcome in selected studies with preoperative MRI. These findings align with a recent meta‐analysis that identified horseshoe type and multiple tracts as among the risk factors for recurrence, though the estimates were not derived from MRI data.^37^ Adding these parameters to the radiology report and using them to assess the likelihood of future recurrence or healing can be reassuring to patients. Additionally, the presence of fistula tract on postoperative MRI, healing of internal opening, healing of intersphincteric tract, healing of external tracts, and the development of new abscess was found to affect the healing process in the four studies that investigated the impact of postoperative MRI.

MRI is superior to both endosonography and examination under anesthesia to delineate the anatomy of AF such as the sphincters, pelvic muscles, and tract course. It can effectively detect inflammation, fibrosis, and healing of the fistula tract.^38^, ^39^ Moreover, the review of MRI scans by the operating surgeon can lead to alterations in the treatment plan, further emphasizing its importance in the management of AF and its superiority to other modalities. Both preoperative and postoperative MRI scans can be useful in predicting patient outcomes, with significant overlap in their findings. However, preoperative MRI allows for consultation with the patient before surgery, leading to increased compliance and improved patient satisfaction. Preoperative MRI is generally reserved for complex cases and is used to assess the complexity of the fistula and determine the most appropriate surgical approach or technique.^40^ On the other hand, postoperative MRI is typically performed in cases where complications arise after surgery, such as recurrence, development of a new abscess, or persistent pain.^41^ It is important to note that the inflammatory response following surgery can complicate the interpretation of postoperative MRI scans, and therefore, radiologists should have a thorough understanding of the subject. Accordingly, some experts suggest that acquiring postoperative MRI after 12 weeks of surgery is particularly useful for assessing healing and detecting complications.^42^ To the best of our knowledge, sensitivity analysis has not been conducted to evaluate the effects of preoperative versus postoperative MRI, likely due to the recent application of postoperative MRI in AF patients.

This review had several limitations. We did not conduct a meta‐analysis due to significant heterogeneity between the included studies. The heterogeneity was primarily related to differences in interventions, their success rates, predictors, and outcome measures across the studies. These differences made it challenging to combine the data and draw meaningful conclusions. The follow‐up duration in some patients was less than 3 months, and it does not seem practical to evaluate the outcome in these patients. In addition, various interventions and different settings may have affected the overall outcome, since the success rate is affected by the surgeon's expertise and vary between different procedures. Thus, the current study highlights the need for more high‐quality prospective studies to better understand the role of MRI in the management of AF. Future studies should focus on larger patient populations, longer follow‐up periods, and the use of standardized outcome measures to allow for more robust comparisons across studies.

## 5 CONCLUSION

In summary, this review revealed that several factors, including fistula length, horseshoe type, presence of multiple tracts, supralevator extension, and ADC value, were significantly associated with the reported outcome. In contrast, the impact of postoperative MRI on AF outcome was less studied, but the presence of the fistula tract and the development of new abscess were found to hinder the healing process. These findings highlight the potential usefulness of MRI in the management of AF, both preoperatively and postoperatively, and provide insight into factors that may influence treatment outcomes. Further studies are needed to confirm these findings and to determine the optimal use of MRI in the management of AF.

## AUTHOR CONTRIBUTIONS

**Alireza Teymouri**: Conceptualization; methodology; writing—original draft; writing—review and editing. **Amir Keshvari**: Data curation; formal analysis. **Ali Ashjaei**: Investigation; supervision; validation. **Seyed Mohsen Ahmadi Tafti**: Conceptualization; data curation; validation. **Faeze Salahshour**: Methodology; validation; writing—review and editing. **Faezeh Khorasanizadeh**: Conceptualization; validation; writing—review and editing. **Amirhosein Naseri**: Project administration; supervision; validation.

## CONFLICT OF INTEREST STATEMENT

The authors declare no conflict of interest.

## TRANSPARENCY STATEMENT

The lead author Amirhosein Naseri affirms that this manuscript is an honest, accurate, and transparent account of the study being reported; that no important aspects of the study have been omitted; and that any discrepancies from the study as planned (and, if relevant, registered) have been explained.

## Data Availability

The authors confirm that this article's data are available within the article.

## 1

FULL SEARCH TERMS FOR PUBMED, EMBASE, AND EBSCO DATABASES

PubMed

(“Rectal Fistula”[MeSH Terms] OR “Anal fistula”[Text Word] OR “Rectal Fistula”[Text Word] OR “fistula in ano”[Text Word] OR “perianal fistula”[Text Word] OR “anus fistula”[Text Word] OR “anorectal fistula”[Text Word] OR “fistulotomy”[Text Word] OR “fistulectomy”[Text Word] OR “advancement flap”[Text Word] OR “seton”[Text Word] OR “anal fistula surgery”[Text Word]) AND (“Recurrence”[MeSH Terms] OR “recur*”[Text Word] OR “relaps*”[Text Word] OR “persist*”[Text Word] OR “outcome*”[Text Word] OR “healing”[Text Word] OR “non‐healing”[Text Word] OR “failure”[Text Word] OR “Treatment Outcome”[MeSH Terms] OR “Treatment Failure”[MeSH Terms]) AND (“Magnetic Resonance Imaging”[MeSH Terms] OR “MRI”[Text Word] OR “Magnetic Resonance Imaging”[Text Word] OR “magnetic”[Text Word] OR “MR imaging”[Text Word])

Embase

(‘anus fistula’/exp OR ‘anus fistula’:ab,ti OR ‘anorectal fistula’:ab,ti OR ‘perianal fistula’:ab,ti OR ‘fistula in ano’:ab,ti OR ‘rectum fistula’:ab,ti OR fistulotomy:ab,ti OR fistulectomy:ab,ti OR ‘advancement flap’:ab,ti OR seton:ab,ti) AND (‘nuclear magnetic resonance’/exp OR ‘nuclear magnetic resonance imaging’/exp OR mri:ab,ti OR ‘magnetic resonance imaging’:ab,ti OR magnetic:ab,ti OR ‘mr imaging’:ab,ti) AND (‘recurrent disease’/exp OR ‘persistence’/exp OR recur*:ab,ti OR relaps*:ab,ti OR ‘treatment outcome’:ab,ti OR ‘treatment failure’:ab,ti)

EBSCO

(SU “Anal fistula” OR AB “fistula in ano” OR AB “anorectal fistula” OR AB “perianal fistula” OR AB “anus fistula” OR AB “rectal fistula” OR AB fistulotomy OR AB fistulectomy OR AB “advancement flap” OR AB seton) AND (SU “Magnetic resonance imaging” OR AB MRI OR AB “MR imaging” OR AB “Magnetic resonance imaging” OR AB magnetic) AND (SU recurrence OR SU “treatment outcome” OR SU “treatment failure” OR AB recur* OR AB relaps* OR AB outcome* OR AB fail* OR AB healing OR AB “non‐healing”)
